# Vulnerability of Newborns to Environmental Factors: Findings from Community Based Surveillance Data in Bangladesh

**DOI:** 10.3390/ijerph8083437

**Published:** 2011-08-22

**Authors:** Ishtiaq Mannan, Yoonjoung Choi, Anastasia J. Coutinho, Atique I. Chowdhury, Syed Moshfiqur Rahman, Habib R. Seraji, Sanwarul Bari, Rasheduzzaman Shah, Peter J. Winch, Shams El Arifeen, Gary L. Darmstadt, Abdullah H. Baqui

**Affiliations:** 1 Department of International Health, Johns Hopkins Bloomberg School of Public Health, 615 N. Wolfe Street, Suite E8138, Baltimore, MD 21205, USA; E-Mails: ychoi@usaid.gov (Y.C.); anastasia.coutinho@gmail.com (A.J.C.); mohshah@jhsph.edu (R.S.); pwinch@jhsph.edu (P.J.W.); gdarmsta@jhsph.edu (G.L.D.); abaqui@jhsph.edu (A.H.B.); 2 Save the Children, H 1(A) 2, Road 91, Dhaka 1212, Bangladesh; 3 International Center for Diarrhoeal Disease Research, Bangladesh, Mohakhali, Dhaka 1212, Bangladesh; E-Mails: atiquei@icddrb.org (A.I.C.); moshfiq@icddrb.org (S.M.R.); habibur.seraji@adelaide.edu.au (H.R.S.); bari@icddrb.org (S.B.); shams@icddrb.org (S.E.A.)

**Keywords:** neonatal, infection, sepsis, community health workers, environment, heat humidity index, Bangladesh

## Abstract

Infection is the major cause of neonatal deaths. Home born newborns in rural Bangladeshi communities are exposed to environmental factors increasing their vulnerability to a number of disease agents that may compromise their health. The current analysis was conducted to assess the association of very severe disease (VSD) in newborns in rural communities with temperature, rainfall, and humidity. A total of 12,836 newborns from rural Sylhet and Mirzapur communities were assessed by trained community health workers using a sign based algorithm. Records of temperature, humidity, and rainfall were collected from the nearest meteorological stations. Associations between VSD and environmental factors were estimated. Incidence of VSD was found to be associated with higher temperatures (odds ratios: 1.14, 95% CI: 1.08 to 1.21 in Sylhet and 1.06, 95% CI: 1.04 to 1.07 in Mirzapur) and heat humidity index (odds ratios: 1.06, 95% CI: 1.04 to 1.08 in Sylhet and, 1.03, 95% CI: 1.01 to 1.04 in Mirzapur). Four months (June–September) in Sylhet, and six months in Mirzapur (April–September) had higher odds ratios of incidence of VSD as compared to the remainder of the year (odds ratios: 1.72, 95% CI: 1.32 to 2.23 in Sylhet and, 1.62, 95% CI: 1.33 to 1.96 in Mirzapur). Prevention of VSD in neonates can be enhanced if these interactions are considered in health intervention strategies.

## 1. Introduction

More than one-third and one-quarter of the four million neonatal deaths each year worldwide are estimated to be due to severe infection and sepsis, respectively [1,2]. The majority of these deaths are in developing countries, where home birth continues to be a norm and, unprotected against the environment in poor rural households, newborns are likely to be more vulnerable compared to older infants and adults [3].

A wide range of morbidity and mortality has been shown to be influenced by the external environment, including changes in temperature, humidity, and rainfall throughout seasons. Previous studies have reported seasonal variation in the incidence of birth weight [4–8], preterm births [9–12], hypothermia [3,13], viral and upper respiratory infections [3,14–21], pneumonia [22], typhoid [23], skin infections [3,21], eye infections [3,21], encephalitis [22], meningitis [24], umbilical infections [3], malaria [25–27], cholera [28,29], and non-cholera diarrhea [22,30–41]. Focused on older age groups, the commonality of these hospital-based studies, where environmental factors are regulated to a certain extent, leave limited data on the seasonal variation of morbidities in newborns in the community. To our knowledge, a study in rural Gadchiroli district of India is the only study that specifically examined the association of newborn morbidities with specific environmental factors in a community setting [3], and none has reported seasonal variation of newborn sepsis.

Countries in South Asia have adopted national strategies to strengthen management of serious neonatal illness, including serious infection, at the community level [42,43]. These strategies, typically involving community-based surveillance for serious illness and subsequent management, are resource intensive [44,45]. Understanding environmental factors associated with serious newborn illness will provide programmatically useful information to develop strategies for health education, surveillance, and preparedness. This study aimed to assess the association of serious newborn illness in the community with three environmental factors: temperature, rainfall, and humidity. We attempt to establish a programmatically relevant seasonality pattern, based on the levels of environmental factors and discuss potential causal and contributing pathways that explain the epidemiology presented.

## 2. Methods

### 2.1. Study Setting

**Figure 1.**
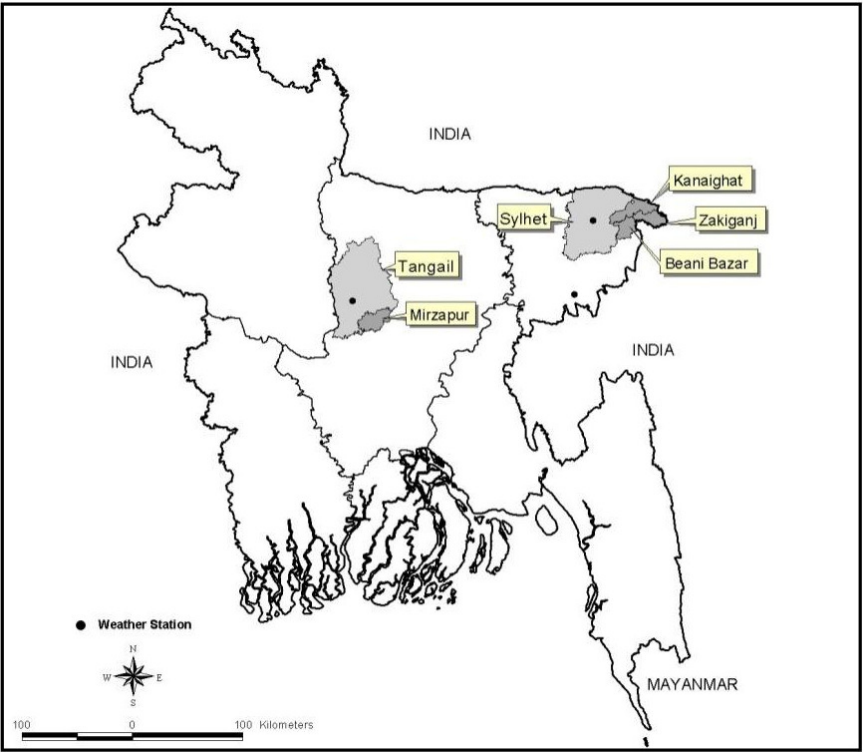
Bangladesh map showing study areas and meteorological stations.

This study uses data from two cluster-randomized controlled trials, known as Projahnmo-1 and Projahnmo-2, conducted during 2004–2006 in Sylhet district and Mirzapur sub-district, respectively, that assessed the effectiveness of community-based intervention packages to improve maternal and newborn care practice and care-seeking. Located in the northeast of Bangaldesh, the Sylhet study area had approximately 500,000 population, and Mirzapur, in central Bangladesh, had approximately 300,000 population (Figure 1). Details of the study population, study design, and interventions have been described elsewhere [44,46–48]. Community health workers (CHWs) in the home-care arm of Projahnmo-1 and the intervention arm of Projahnmo-2, each covering a population of about 4,000, conducted active surveillance for serious newborn illness. CHWs made routine postnatal home visits in Sylhet (on days 0, 2 and 6) and Mirzapur (on days 0, 2, 5, and 8) using a sign-based algorithm adapted from the Bangladesh Integrated Management of Childhood Illness (IMCI) guidelines. CHWs also educated family members on newborn danger signs and requested that families call them to assess any infant who developed danger signs in the neonatal period. CHWs in both areas completed standard assessment forms for presence of selected 28 signs and 16 historic factors of suspected serious illness. Then, CHWs classified neonates with very severe disease (VSD) using clinical algorithms. While Sylhet CHWs used an 8-sign algorithm in Mirzapur, three additional signs associated with high case-fatality were incorporated and CHWs used a modified 11-sign algorithm for diagnosis, both algorithms have been validated against physicians’ judgment of neonatal illness needing referral to a hospital [47]. Detailed information on these algorithms is described elsewhere [46,47]. To identify suspected serious illness, Sylhet CHWs used an 8-sign algorithm to classify sick newborns as having very severe disease (VSD).

### 2.2. CHW Training

CHWs were women recruited from the locality with at least 10 years of schooling. Generally, they had no prior training in health, but received initial training for six weeks (including six days of field practice) through didactic sessions, video demonstrations and practice on sick and healthy newborns in Sylhet Medical College Hospital and in Kumudini Hospital in Mirzapur. Their performance was monitored, evaluated and documented throughout the training, and their competence in assessment of newborns at the hospital was confirmed before beginning field work. Field supervisors checked the quality and accuracy of data collection through on-site observation using standard checklists and routinely checked CHWs’ records for internal consistency before they were entered into a database. A sample of newborns was re-assessed by physicians to validate CHWs’ ascertainment of danger signs and classification and management of illness, and documented that CHWs’ classification of newborn illness was highly sensitive and specific.

### 2.3. Data

Data used in the current analysis came from newborn illness surveillance conducted in Projahnmo-1 and Projahnmo-2 [46,47]. Neonatal illness surveillance recorded 10,585 live births in Sylhet in the 24 months between January 2004 and December 2005, and 10,407 live births in Mirzapur in the 36 months between January 2004 and December 2006. A total of 9,370 (88.5%) and 7,877 (75.7%) were born at home in Sylhet and Mirzapur, respectively, and 8,474 (80.1%) newborns in Sylhet and 7,587 (72.9%) neonates in Mirzapur received at least one visit during the entire neonatal period between days 1 and 28. We restricted our analysis to those who were born at home and assessed by CHWs at least once during the routine active surveillance window (*i.e.*, the first seven and nine days of life in Sylhet and Mirzapur, respectively, hereafter referred to as the active surveillance period). In total, 6,936 and 5,900 neonates were analyzed in Sylhet and Mirzapur, respectively. These newborns received on average 2.6 (SD: 0.86, Median: 3.0) assessments in Sylhet and 3.6 (SD: 0.97, Median: 4.0) assessments in Mirzapur during the active surveillance period.

We obtained environmental data from the Bangladesh Meteorological Department. For Sylhet, data were obtained from a meteorological station located at the Sylhet city airport at an average distance of 37.5 km (SD ± 12.4) from study area and, and for Mirzapur, data were obtained from a station located at Tangail Sadar, an average distance of 22.4 km (SD ± 4.8) from the study area (Figure 1). Daily values of minimum and maximum temperature (°C), relative humidity (%) and rainfall (mm) were collected for the entire surveillance period. The Stevenson Screen System was used for recording air temperature, which was collected every three hours. Dry-bulb and wet-bulb thermometers were used to measure humidity and relative humidity was calculated from the difference between dry and wet bulb measures using a hygrometric table. Both an ordinary rain gauge and self-recording rain gauge were used to measure rainfall at 3-hour intervals.

### 2.4. Measurement and Analysis

To measure VSD, we applied the 11-sign VSD algorithm used in Mirzapur to both populations to facilitate interpretation of results across sites. In addition to daily values for environmental factors (mean of the minimum and maximum temperature, relative humidity, and rainfall), we calculated a daily heat humidity index (HHI) as follows: HHI = T − (0.55 − 0.55 × RH) × (T − 58) [12,49], where T is dry bulb temperature in Fahrenheit and RH is percent relative humidity. Then, we calculated a 7-day rolling average for each environmental factor. For newborns with VSD, the average environmental factor values for the seven days prior to the date of identification of VSD were calculated and for healthy newborns the environmental factors values averaged over the seven days prior to the end of active surveillance periods were calculated. This seven-day period was used to allow a latency period between environmental factors and incidence of serious illness. Since there is no standard recommendation, we decided to use seven days after conducting a series of sensitivity analysis by using varying lengths of latency periods—3, 5, and 10 days.

Multivariable logistic regression analysis were conducted to assess associations between environmental factors and the odds of having VSD, controlled for background covariates (sex, parity, preterm, maternal age, maternal education and household building material). We explored three different models, each with a different set of main independent variable(s): mean raw environmental values during the seven days (mean temperature, humidity, and rainfall during the seven days) (Model 1), mean HHI during the seven days (Model 2), and birth month (born during high-risk months or not) (Model 3). High-risk months were those with a mean temperature >28 °C and corresponded to the four months between June and September in Sylhet and the six months between April and September in Mirzapur. A p-value < 0.05 was considered statistically significant. STATA 10.0 statistical software (Stata Corporation, College Station, TX, USA) was used for all analysis.

### 2.5. Ethical Clearance

The studies were approved by the Committee on Human Research at the Johns Hopkins Bloomberg School of Public Health, and the Ethical Review Committee and the Research Review Committee at ICDDR, B. Studies were registered at clinicaltrials.gov, No. 00198705 (Projahnmo-1) and No. 00198627 (Projahnmo-2).

## 3. Results

**Figure 2.**
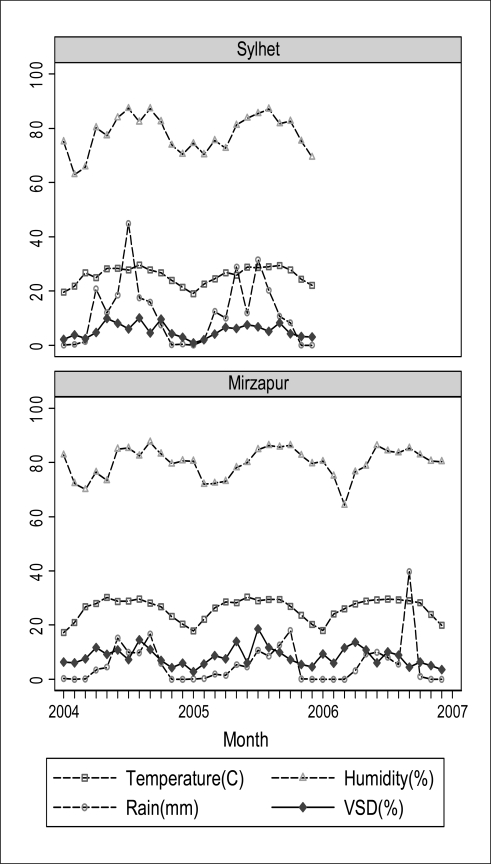
Monthly pattern of environmental factors and percent of neonates with VSD during the active surveillance window: Sylhet 2004–2005 and Mirzapur 2004–2006.

Table 1 provides the monthly averages of environmental factors for 24 months (2004–2005) in Sylhet and 36 months (2004–2006) in Mirzapur. Figure 2 plots these monthly values over the entire surveillance periods along with incidence of very severe disease (VSD) per 100 neonates. Generally, in both sites, May through October was the period when all indices were at their highest levels.

**Figure 3.**
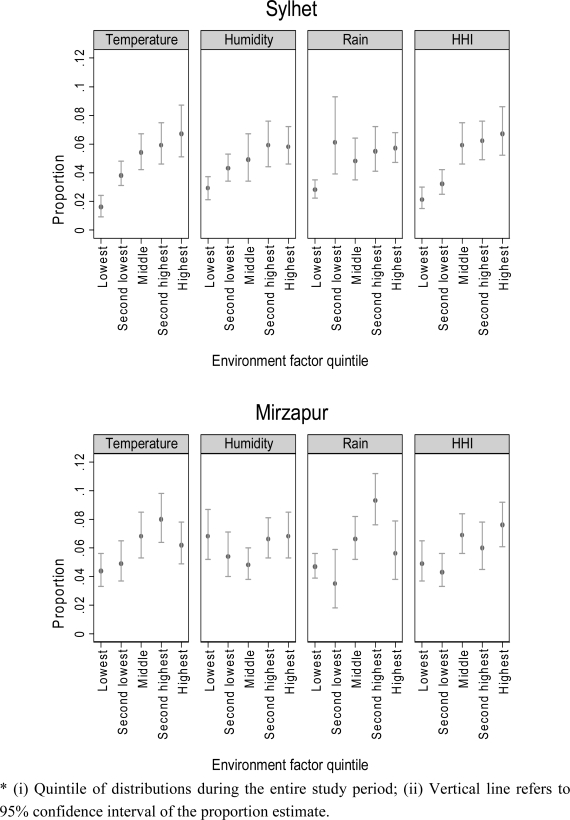
Proportion of neonates with VSD across quintiles of environmental factors *. * (i) Quintile of distributions during the entire study period; (ii) Vertical line refers to 95% confidence interval of the proportion estimate.

VSD incidences corresponded with temperature and humidity curves in both sites, but not with rainfall (Figure 2). Figure 3 shows the unadjusted proportions of newborns with VSD by quintiles of each environmental variable. In Sylhet, clear linear associations were observed between VSD proportion and each environmental factor, whereas in Mirzapur the association was obvious only for temperature. Adjusted for covariates, however, only temperature was significantly associated with incidence of VSD in both sites; odds ratios (ORs) were 1.14 (95% CI: 1.08 to 1.21) in Sylhet and 1.06 (95% CI: 1.04 to 1.07) in Mirzapur (Table 2—Model 1). For HHI, ORs were 1.06 (95% CI: 1.04 to 1.08) in Sylhet and 1.03 (95% CI: 1.01 to 1.04) in Mirzapur (Table 2—Model 2). In both Sylhet and Mirzapur, newborns born in high-risk months had significantly higher odds of having VSD (ORs: 1.72 (95% CI: 1.32 to 2.23) in Sylhet and 1.62 (95% CI: 1.33 to 1.96) in Mirzapur) (Table 2—Model 3). These months also corresponded to the highest levels of HHI in Sylhet (Table 1).

Table 3 confirms the higher incidence of VSD during the higher-risk, higher-temperature months. We further examined the individual danger signs by higher and lower risk months. Respiratory rates >70 breaths per minute and fever >101 °F were significantly higher in both sites during the high-risk months as compared to the rest of the year.

## 4. Discussion

To our knowledge, this is one of the very few studies to show variations in very severe disease (VSD) in rural communities in the context of environmental factors. We explored seasonality of VSD by specific measures of environmental factors and found that, unadjusted, temperature, rainfall, humidity, and HHI levels were correlated with incidence of VSD in newborns delivered in rural homes. When adjusted for other background factors, temperature alone and HHI, largely driven by temperature, were found to be associated with incidence of VSD; however, the size of the effect was relatively small. Four consecutive months of the year in Sylhet and six in Mirzapur, with mean temperature levels above 28 °C and higher HHI, had significantly higher odds of incidence of VSD compared to the rest of the year, although the contribution of other confounding factors that were not controlled for in the analysis is unknown. Specific signs of severe disease, *i.e.*, high respiratory rate and fever, were more common in these high-risk months.

As one of the very few studies examining the relationship between environmental factors and VSD in a community context, the high coverage of active surveillance for illness and daily measures of environmental factors over the entire surveillance period of three years provides a finer temporal resolution for correlating changes in environmental factors with incidence of VSD. However, this may mean the statistical associations found between temperature and humidity with odds of severe neonatal illness may have little practical relevance. Application of our findings to programmatic settings should be made with caution, since VSD associated with high case fatality occurs throughout the year and thus requires careful vigilance by the families and the health systems for every newborn. However, seasonal variation in illness was strong, and may be, in part, modulated by temperature and humidity, but likely involves other factors unaccounted for in this analysis.

Our findings should be interpreted considering the limitations that we used household building materials as proxy for a comprehensive wealth index and could not control for birth weight in the regression model. By study design, birth weight was collected in only one site and the time for measurement varied due to logistical constraints of reaching newborns at the earliest possible time. However, we controlled for gestational age.

Few studies have examined seasonal variations of sepsis occurrence; most examined sepsis in adolescents and adults [50–53] and only one study was conducted in a developing country [50]. Contrary to our findings in neonates in a tropical climate, one study conducted in England [53] and two studies conducted in the United States [51,52] in temperate climates showed increases in sepsis cases in adults in the winter months, which Danai *et al*. attributed to increases in sepsis incidence occurring from an initial respiratory source [51]. Other studies have shown that seasonality of disease and environmental fluctuations differ by latitude [35,54,55], although this has not been examined for sepsis specifically. The SEARCH trial in Gadchiroli, with closest similarity with our settings showed no seasonal variation of suspected neonatal sepsis, but found higher incidences of hypothermia, upper respiratory symptoms, umbilical and bacterial skin infections in winter, and of unexplained fever in summer [3]. Bang *et al*. suggested that lack of protection from the effects of the environment played a major role in the seasonal patterns observed [3].

Association of serious neonatal illness, and specifically serious neonatal infections, with environmental factors in a rural developing country setting can potentially be explained under two pathways, often sequential or overlapping: (1) pathogenic (bacterial or viral) and (2) behavioral. In both of our sites, VSD was found to be associated with increased temperature levels. In warmer and more humid months, it is likely that a variety of pathogens proliferate in the environment, as demonstrated for *Klebsiella pneuminiae*, a common source of community-acquired infections [56]. An increase in growth may lead to an increase in virulence or inoculums [57], as well as colonization. The proposition that bacterial growth increases with increasing temperature and humidity is supported by numerous other studies [29,57–61]. However, there are also controversies around the influence of temperature and humidity on bacterial pathogens. Laboratory studies show considerable differences on the effect of temperature or humidity on bacteria strains [62–67], indicating that many factors outside of environmental causes are involved in disease transmission. Behavioral patterns are seasonal and could have contributed to contamination and infection transmission. Lack of ventilation in rooms during the summer, which is very common in rural Bangladesh, as well as an increase in the number of individuals indoors during the monsoon season, might have resulted in overcrowding and the transmission of pathogens [3,18,59]. During the summer, neonates might have more contact with others and use less clothing, decreasing protection from the environment and increasing the spread of transmission [68].

## 5. Conclusions

The observed seasonality of serious neonatal illness indicates that changes in environmental factors may modulate risk for serious illness. These environmental factors may serve as proxies for other proximate factors in health (e.g., bacterial growth, nutritional and growth status, amount of labor, social and household behaviors and practices, *etc*.), and thus, cause and effect have not been clearly delineated. Future studies should examine these factors. Health education strategies need to promote preventive measures addressing behavioral issues to minimize effects of environment on newborns throughout the year, more so during the high-risk months. Program planning processes should take seasonality into account and apply that for projecting case loads and align intervention delivery strategies accordingly.

## Figures and Tables

**Table 1.** Monthly average of temperature, rainfall, humidity and heat humidity index (HHI) by site.

	**Sylhet (2004–2005)**				**Mirzapur (2004–2006)**			

**Months**	**Temperature [°C]**	**Rain [mm]**	**Humidity [%]**	**Heat-Humidity Index (HHI)**	**Temperature [°C]**	**Rain [mm]**	**Humidity [%]**	**Heat-Humidity Index (HHI)**
January	19.3	0.0	74.8	65.5	17.7	0.2	81.2	63.3
February	22.2	1.0	66.6	69.4	22.3	0.1	73.1	70.0
March	25.6	6.9	70.7	74.8	26.4	0.7	68.9	76.0
April	25.9	15.3	76.5	76.0	28.1	2.7	75.3	79.2
May	27.1	20.4	79.2	78.2	29.2	6.4	76.7	81.2
June	28.6	15.1	83.9	81.2	29.5	9.9	83.7	82.7
July	28.2	38.3	86.5	80.9	29.1	9.5	84.8	82.2
August	29.3	18.9	84.8	82.5	29.5	7.9	84.1	82.7
September	28.6	13.3	84.5	81.3	28.9	23.0	86.2	82.0
October	27.1	8.0	82.6	78.6	27.3	8.2	84.1	79.0
November	24.2	0.1	74.6	73.1	23.6	0.0	80.9	72.7
December	21.8	0.2	69.9	69.0	20.2	0.0	80.2	67.2
Mean (sd)	25.7 (±3.2)	11.5 (±11.3)	77.9 (±6.8)	75.9 (±5.6)	26.0 (±4.0)	5.7 (±6.8)	79.9 (±5.3)	76.5 (±6.7)
Range	19.3, 29.3	0.0, 38.3	66.6, 86.5	65.5, 82.5	17.7, 29.5	0.0, 23.0	68.9, 86.2	63.3, 82.7

**Table 2.** Differential odds of having VSD during the routine postnatal visit window * in Sylhet and Mirzapur by environmental factors: multivariable regression analysis **.

	**Model 1**		**Model 2**		**Model 3**	
	**Odds Ratio**	**P value**	**Odds Ratio**	**P value**	**Odds Ratio**	**P value**
**Sylhet**						
Temperature	1.14 (1.08, 1.21)	0.00				
Humidity	1.01 (0.99, 1.02)	0.32				
Rainfall	1.00 (0.98, 1.02)	0.99				
Heat Humidity						
Index (HHI)			1.06 (1.04, 1.08)	0.00		
High Risk Month						
(June–August)					1.72 (1.32, 2.23)	0.00
**Mirzapur**						
Temperature	1.06 (1.04, 1.07)	0.00				
Humidity	1.00 (0.98, 1.03)	0.83				
Rainfall	1.00 (0.99, 1.00)	0.48				
Heat Humidity						
Index (HHI)			1.03 (1.01, 1.04)	0.00		
High Risk Month						
(April–September)					1.62 (1.33, 1.96)	0.00

* First 7 days in Sylhet and first 9 days in Mirzapur;

** Adjusted for sex, preterm birth, parity, maternal age, women education, household building materials.

**Table 3.** Incidences of VSD and individual danger signs by high- and low-risk months by sites.

	**Sylhet**			**Mirzapur**		

	**Overall n = 6936 (%)**	**High-risk month n = 2057 (%)**	**Low-risk month n = 4879 (%)**	**Overall n = 5900 (%)**	**High-risk month n = 2542 (%)**	**Low-risk month n = 3358 (%)**
VSD	309 (4.5)	128 (6.2) *	181 (3.7)	352 (6.0)	193 (7.6) *	159 (4.7)
Convulsion	23 (0.3)	11 (0.5)	12 (0.3)	10 (0.2)	8 (0.3)	2 (0.1)
Respiratory rate 70/min or higher	115 (1.7)	54 (2.6) *	61 (1.3)	84 (1.4)	61 (2.4) *	23 (0.7)
Severe chest in-drawing	17 (0.3)	9 (0.4)	8 (0.2)	11 (0.2)	6 (0.2)	5 (0.2)
Temperature > 101 °F	24 (0.4)	17 (0.8) *	7 (0.1)	19 (0.3)	16 (0.6) *	3 (0.1)
Temperature < 95.5 °F	84 (1.2)	23 (1.1)	61 (1.3)	84 (1.4)	24 (0.9)	60 (1.8) *
Unconsciousness	7 (0.1)	5 (0.2)	2 (0.0)	1 (0.0)	0 (0.0)	1 (0.0)
Skin problems	23 (0.3)	11 (0.5)	12 (0.3)	54 (0.9)	29 (1.1)	25 (0.7)
Umbilical redness extending to skin	9 (0.1)	4 (0.2)	5 (0.1)	10 (0.2)	5 (0.2)	5 (0.2)
Week, abnormal or absent cry	64 (0.9)	22 (1.2)	42 (0.9)	48 (0.8)	27 (1.2)	21 (0.6)
Lethargic, less than normal movement	91 (1.3)	33 (1.6)	58 (1.2)	83 (1.4)	43 (1.7)	40 (1.2)
Not able to feed or not suck at all	94 (1.4)	30 (1.5)	64 (1.3)	123 (2.1)	58 (2.3)	65 (1.9)

* Chi-squared p-value < 0.01
